# LATE-LIFE CARE FOR OLDER ADULTS: FINDINGS FROM NHATS AND NSOC

**DOI:** 10.1093/geroni/igac059.1640

**Published:** 2022-12-20

**Authors:** Mengyao Hu, Sarah Patterson

## Abstract

With the aging of the U.S. population, caregiving has become an emerging public health issue that affects the health and quality of life for millions of care recipients and their caregivers. An increasing number of older adults rely on their family caregiver networks and home-based clinical services for physical, social and emotional support. Caregiving plays an important role in health and wellbeing of older adults both in daily life and in the context of health care, e.g., in post-acute caregiving after hospital discharge. There is a growing interest in identifying caregivers in greatest need of support and developing programs and interventions to help these caregivers. This symposium describes caregiver network and examines the roles of the caregiver network and family caregiving support on care recipients’ and caregivers’ quality-of-life outcomes using the National Health and Aging Trends Study (NHATS) and linked National Study on Caregiving (NSOC). This symposium will 1) evaluate typologies of the structures and compositions of caregiver network and examine their effects on care recipients’ well-being; 2) describe findings on associations between caregiving network and caregiver supports with unmet needs among older adults; 3) describe the role of unpaid caregivers after hospital discharge; 4) evaluate effects of family caregiving support in facilitating the use of home-based clinical services by older adults; 5) examine the effects of family disagreement on caregivers’ emotional difficulty and overload in dementia caregiving. Together, these presentations suggest important public health implications for research, policy and practice for improving late-life caregiving.

